# The Week's Parliament and Publications

**Published:** 1910-07-30

**Authors:** 


### The Week's Parliament and Publications.

### ADULTERATION OF FLOUR.

Mr. Broadley has again drawn the attention of the Secretary of the Local Government Board to the addition to genuine flour of a so-called improver. The hon. member states that some of these " improvers " contain arsenic, and many of them are simply preparations of purified bone ash, while others contain large quantities of gypsum or sulphate of lime. The points raised by Mr. Broadley are receiving attention in connection with the inquiry which Mr. Burns has directed into the bleaching of flour.

### THE DUTY ON SPIRIT.

In reply to a question asked in the House of Commons by Mr. Primrose, as to whether the Chancellor of the Exchequer would be willing to allow a drawback of the duty on spirit used by medical practitioners and chemists and druggists, Mr. Lloyd George replied that he has given his sympathetic consideration to this matter; the practical difficulties are very serious, however, and he is afraid that he cannot undertake to grant such a drawback. This question was dealt with in The Hospital of July 9, page 445.

### DISPARITY IN THE VALUE OF MEDICAL DEGREES.

In the House of Commons on Monday Mr. Lynch asked the Home Secretary whether his attention had been called to the disparity in the value of medical degrees and diplomas which entitled their possessors to practise medicine in this country; and whether he would take steps by legislation or otherwise towards establishing a fair standard of examination qualifying for medical practice. In his reply Mr. Churchill stated that it was the duty of the General Council of Medical Education to inspect and report on all qualifying examinations, and if it appeared to them that the standard of proficiency required at the qualifying examinations held by any of the licensing bodies was insufficient, to make a representation to that effect to the Privy Council, who are empowered, if they think fit after due inquiry, to order the withdrawal from medical authorities of the right to hold qualifying examinations.

### MIDWIVES BILL.

The Midwives (No. 2) Bill has been read a second time in the House of Lords. Lord Clonbrock has intimated that in committee he will move an amendment with the object of bringing Ireland within the scope of the Bill.

### MEDICAL PRACTITIONERS AND THE PETROL TAX.

In answer to a question asked by Mr. Courthope, the Chancellor of the Exchequer replied that the Commissioners of Customs and Excise will be prepared, for the present, to consider claims for repayment of petrol duty in the case of medical practitioners owning cars for professional purposes.

### INSTRUCTION IN HYGIENE.

Dr. Addison has introduced a Bill in the House of Commons to require that in public elementary schools instruction shall be given in hygiene, and to girls in the care and feeding of infants. In introducing the Bill, Dr. Addison said that something like one-third of the waste of child-life was due to improper feeding and ignorance on the part of those who had the care of children.

### MEDICAL INSPECTION OF SCHOOL CHILDREN.

In introducing the Board of Education estimates, Mr. Runciman gave some interesting particulars concerning the medical inspection of school children. Every one of the local authorities has now undertaken this work, and over 1,500,000 children have been examined. One thousand medical officers have been appointed, and there are 300 school nurses. It has been found that there are large numbers of parents who are unwilling or unable to provide the treatment necessary for their children, and such cases are met in a variety of ways. Some authorities pay for nursing assistance out of the Education rate. Up to the present month 65 authorities have done this; 33 of them are paying for spectacles out of the rates; 15 of them are contributing to hospitals out of the rates, and ten of them have established school crèches.

